# Tumor burden of persistent disease in patients with differentiated thyroid cancer: correlation with postoperative risk-stratification and impact on outcome

**DOI:** 10.1186/s12885-020-07269-3

**Published:** 2020-08-14

**Authors:** Renaud Ciappuccini, Natacha Heutte, Audrey Lasne-Cardon, Virginie Saguet-Rysanek, Camille Leroy, Véronique Le Hénaff, Dominique Vaur, Emmanuel Babin, Stéphane Bardet

**Affiliations:** 1 Department of Nuclear Medicine and Thyroid Unit, François Baclesse Cancer Centre, 3 Avenue Général Harris, F-14000 Caen, France; 2 INSERM 1086 ANTICIPE, Caen University, Caen, France; 3 CETAPS EA 3832, Rouen University, Rouen, France; 4 Department of Head and Neck Surgery, François Baclesse Cancer Centre, Caen, France; 5 Department of Pathology, François Baclesse Cancer Centre, Caen, France; 6 Department of Oncology, François Baclesse Cancer Centre, Caen, France; 7 Department of Cancer Biology and Genetics, François Baclesse Cancer Centre, Caen, France; 8 Department of Head and Neck Surgery, University Hospital, Caen, France

**Keywords:** Differentiated thyroid cancer, Tumor burden, Risk-stratification, Radioiodine, ^18^FDG PET/CT

## Abstract

**Background:**

In patients with differentiated thyroid cancer (DTC), tumor burden of persistent disease (PD) is a variable that could affect therapy efficiency. Our aim was to assess its correlation with the 2015 American Thyroid Association (ATA) risk-stratification system, and its impact on response to initial therapy and outcome.

**Methods:**

This retrospective cohort study included 618 consecutive DTC patients referred for postoperative radioiodine (RAI) treatment. Patients were risk-stratified using the 2015 ATA guidelines according to postoperative data, before RAI treatment. Tumor burden of PD was classified into three categories, i.e. very small-, small- and large-volume PD. Very small-volume PD was defined by the presence of abnormal foci on post-RAI scintigraphy with SPECT/CT or ^18^FDG PET/CT without identifiable lesions on anatomic imaging. Small- and large-volume PD were defined by lesions with a largest size < 10 or ≥ 10 mm respectively.

**Results:**

PD was evidenced in 107 patients (17%). Mean follow-up for patients with PD was 7 ± 3 years. The percentage of large-volume PD increased with the ATA risk (18, 56 and 89% in low-, intermediate- and high-risk patients, respectively, *p* < 0.0001). There was a significant trend for a decrease in excellent response rate from the very small-, small- to large-volume PD groups at 9–12 months after initial therapy (71, 20 and 7%, respectively; *p* = 0.01) and at last follow-up visit (75, 28 and 16%, respectively; *p* = 0.04). On multivariate analysis, age ≥ 45 years, distant and/or thyroid bed disease, small-volume or large-volume tumor burden and ^18^FDG-positive PD were independent risk factors for indeterminate or incomplete response at last follow-up visit.

**Conclusions:**

The tumor burden of PD correlates with the ATA risk-stratification, affects the response to initial therapy and is an independent predictor of residual disease after a mean 7-yr follow-up. This variable might be taken into account in addition to the postoperative ATA risk-stratification to refine outcome prognostication after initial treatment.

## Background

In patients with differentiated thyroid cancer (DTC), the risk-stratification system described in the 2015 American Thyroid Association (ATA) guidelines is a useful tool to predict the likelihood of postoperative persistent disease (PD), the response to initial therapy (i.e. surgery ± radioiodine [RAI] treatment) and the long-term outcome [1]. Several features related to PD are likely to influence the response to treatment and the long-term prognosis. This includes the location of PD (neck lymph-nodes [LN] or distant metastases), the RAI-avidity [2] or ^18^F-Fluorodeoxyglucose (^18^FDG)-avidity [3] of PD, the aggressiveness of pathological variants [4] and the degree of cell-differentiation [5], the presence of molecular mutations (BRAF, TERTp) [6] and the tumor doubling-time [7]. Alone or in combination with previous characteristics, notably RAI-avidity, the tumor burden of PD is another variable that can affect treatment efficiency and prognosis. This has been shown in studies, sometimes old and using low-resolution imaging methods, focusing on patients with distant metastases [2, 8]. In the daily practice, it is well known that microscopic RAI-avid lesions are more likely cured than macroscopic ones, e.g. lung miliary vs. lung macronodules. However, no studies have specified the prognostic role of tumor burden, estimated using high-resolution imaging techniques, both in the setting of distant metastases and lymph-node disease.

The aim of the study was to assess the correlation of PD tumor burden with the 2015 ATA risk-stratification system and its impact on response to initial therapy and outcome. We hypothesized that patients presenting postoperatively a low tumor burden of PD would have better response to initial therapy and better clinical outcomes than patients having high tumor burden.

## Methods

### Patients

The records of 618 consecutive patients with DTC referred to our institution for postoperative RAI treatment between January 2006 and February 2016 were reviewed. For the purpose of the study, patients were risk-stratified according to the 2015 ATA guidelines based on pathological and surgical data available after total thyroidectomy and before postoperative RAI treatment (postoperative risk stratification) [1]. Data available in the preoperative period such as imaging studies showing distant metastases were also used to inform ATA risk stratification. In contrast, postoperative serum thyroglobulin (Tg) level was not used to drive RAI treatment in these patients managed before 2016, and no diagnostic RAI scintigraphy was performed before RAI treatment.

### Postoperative RAI treatment

All 618 patients were administered an RAI regimen 11 ± 7 weeks after total thyroidectomy. Patients were prepared after either thyroid hormone withdrawal (THW) or after two i.m. injections of recombinant human thyrotropin (rhTSH) (Thyrogen, Genzyme Corp., Cambridge, MA, USA), as previously described [9]. TSH level was measured the day of RAI treatment and was > 30 mUI/l in all patients. The RAI activity (1.1 or 3.7 GBq) and the preparation modalities were decided by our multidisciplinary committee. All patients underwent a post-RAI scintigraphy combining whole-body scan (WBS) and neck and thorax single photon emission computed tomography with computed tomography (SPECT/CT). A complementary SPECT/CT (such as abdomen and/or pelvis acquisition) was performed in case of equivocal or abnormal RAI foci on WBS. Patients were scanned two or file days following 1.1 or 3.7 GBq, respectively. Initial therapy was defined as surgery (i.e. thyroidectomy ± LN dissection) plus first RAI treatment (i.e. postoperative RAI treatment).

### Serum Tg and anti-Tg antibodies (TgAb) assay

Blood samples for stimulated serum Tg and TgAb measurements were collected immediately before the RAI treatment. Serum Tg measurements were obtained with the Roche Cobas 6000 Tg kit (Roche Diagnostics, Mannheim, Germany), with a lower detection limit of 0.1 ng/ml and a functional sensitivity of 1.0 ng/ml until October 2013 and with the Roche Elecsys Tg II kit (Roche Diagnostics, Mannheim, Germany), with a lower detection limit of 0.04 ng/ml and a functional sensitivity of 0.1 ng/ml thereafter. TgAb was measured using quantitative immunoassay methods (Roche Diagnostics, Mannheim, Germany). TgAb positivity was defined by the cut-offs provided by the manufacturer.

### Pathology

Pathological variants were defined according to the World Health Organization classification [10]. Poorly differentiated carcinoma, widely invasive follicular carcinoma, Hürthle cell carcinoma, and among PTC variants, tall cell, columnar cell, diffuse sclerosing and solid variants, were considered as aggressive pathological subtypes [1]. Tumor extent was specified according to the TNM 2017 [11].

### Tumor burden of persistent disease

As previously described [9], PD was defined as evidence of tumor in the thyroid bed, LN or distant metastases after completion of initial therapy. Confirmation was achieved either by pathology or by complementary imaging modalities (neck ultrasound examination [US], post-RAI scintigraphy, ^18^FDG positron emission tomography [PET/CT], CT scan or MRI) and follow-up.

**Fig. 1:**
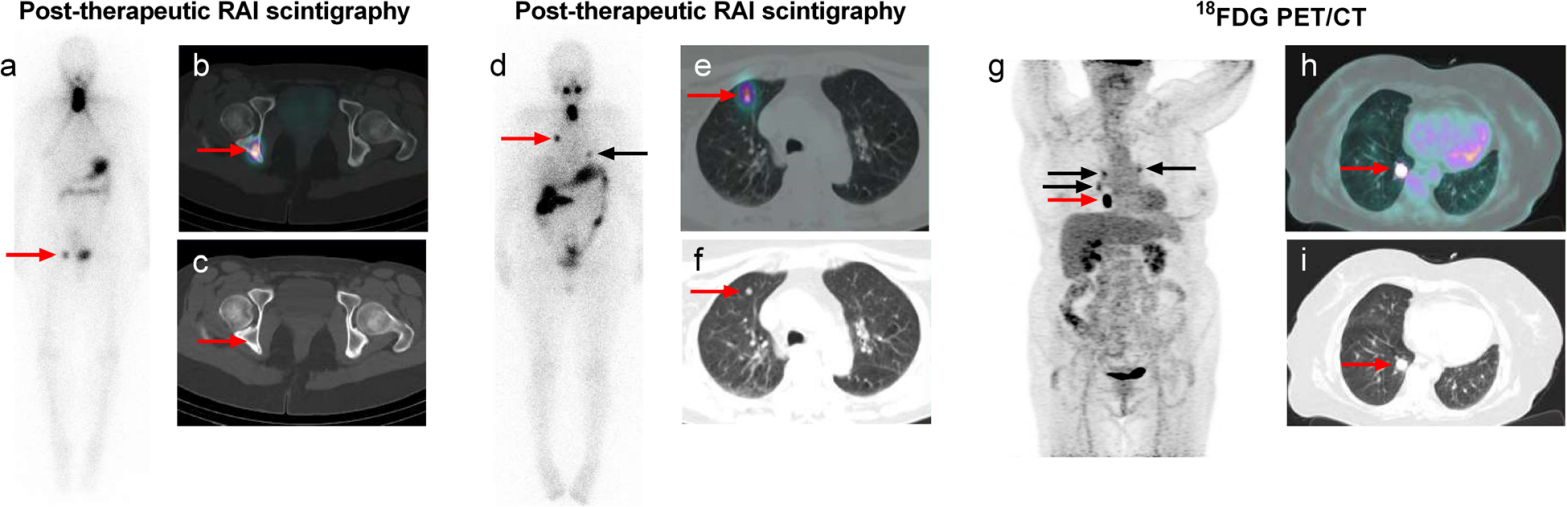
Examples of very small, small and large tumor burden in patients with persistent disease (PD). On the left side, a 43-year-old female patient with a 40-mm PTC at low-risk after initial surgery (T2NxMx) and very small-volume PD (**a**-**c**): post-therapeutic ^131^I WBS showed a solitary bony focus on the right hip (**a**, arrow). Fused transaxial image of ^131^I SPECT/CT (**b**, arrow) confirmed the bony uptake and hybrid CT (**c**, arrow) did not display any bone abnormality. On the middle part, a 74-year-old female patient with a 40-mm PTC at low-risk after initial surgery (T2N0Mx) and small-volume PD (**d**-**f**): post-therapeutic ^131^I WBS showed pulmonary metastases (**d**, red and black arrows). Fused transaxial image (**e**, red arrow) and hybrid CT scan (**f**, red arrow) depicted RAI-avid lung micronodules (**e**-**f**: 6 mm). On the right side, an 88-year-old female patient with a 40-mm PTC (tall cell variant) at high-risk after initial surgery (T2N1bM1) and large-volume PD (**g**-**i**): no abnormal RAI uptake on post-therapeutic ^131^I WBS with SPECT/CT whereas ^18^FDG PET/CT showed pulmonary and mediastinal metastases (**g**, Maximum intensity image, arrows). Fused transaxial image (**h**, arrow) and hybrid CT scan (**i**, arrow) showed high ^18^FDG uptake (SUVmax = 30) by an 18-mm lung nodule.

The tumor burden of PD was classified into three categories, i.e. very small-, small- and large-volume PD. Very small-volume PD was defined by the presence of abnormal foci on post-therapeutic RAI scintigraphy with SPECT/CT or ^18^FDG PET/CT without identifiable lesions on anatomic imaging (neck ultrasound, CT scan or MRI). Small- or large-volume PD were defined by the presence of metastatic lesions with a largest size < 10 or ≥ 10 mm respectively, regardless of RAI or ^18^FDG uptake. Examples of patients with very small-, small-, or large-volume PD are presented in Fig. 1.

### RAI and ^18^FDG uptake in persistent disease

The RAI or ^18^FDG uptake profile was defined at time of PD diagnosis. PD was considered RAI-positive (RAI+) if at least one metastatic lesion showed RAI uptake, and RAI-negative (RAI-) otherwise. Similarly, PD was defined ^18^FDG-positive (^18^FDG+) if at least one metastatic lesion presented significant ^18^FDG uptake, and ^18^FDG-negative (^18^FDG-) otherwise.

### Clinical outcome assessment

As previously described [12], clinical assessment of patients with a negative post-RAI scintigraphy was scheduled at three months with serum TSH, Tg and TgAb measurements while on levothyroxine (L-T4) treatment. When the Tg level at three months was < 1 ng/ml in the absence of TgAb, the disease status was assessed at 9–12 months by serum rhTSH-stimulated Tg assay and neck US, and in recent years, by Tg II assay on L-T4 and neck US. If there was an excellent response at 9–12 months according to the 2015 ATA criteria (i.e. stimulated-Tg level < 1 ng/ml or non-stimulated-Tg level < 0.2 ng/ml without TgAb and negative neck US), patients were followed up on an annual basis. For anything other than an excellent response, imaging modalities such as CT scan of the neck and thorax, ^18^FDG PET/CT or MRI were performed. In case of a second RAI regimen given 6–9 months after the first RAI therapy for RAI-avid PD, post-RAI scintigraphy with SPECT/CT was also used to assess initial treatment response. Responses to initial therapy as assessed at 9–12 months and status at last-visit were categorized as: excellent response, indeterminate response, biochemical incomplete response or structural incomplete response according to the 2015 ATA guidelines [1].

### Data analysis

Quantitative data are presented in mean ± standard deviation (SD), except for Tg levels which are presented in median (range). Patients’ characteristics were compared using Chi-square or Fisher’s exact test, the Wilcoxon test or the Kruskal-Wallis test, as appropriate. The Cochran-Armitage trend test was used to examine proportions of excellent response over the different subgroups in the following order: very-small-, small- and large-volume PD. The analysis of disease-specific survival and progression-free survival was performed using the Cox regression model. The analysis of prognostic factors was performed using logistic regression. Statistical significance was defined as *p* < 0.05. All tests were two-sided. SAS 9.3 statistical software (SAS Institute Inc., Cary, NC, USA) was used for data analysis.

## Results

### Characteristics of patients

The study group included 528 (86%) papillary thyroid cancers (PTC), 63 (10%) follicular thyroid cancers (FTC) and 27 (4%) poorly-differentiated thyroid cancers (PDTC). There were 462 women (75%) and 156 men. The mean age was 50 ± 16 years. Three hundred and seventy-two patients (60%) were prepared with rhTSH stimulation. Eighty-two patients (13%) presented positive TgAb at the time of postoperative RAI treatment. In the postoperative setting prior to RAI administration, 395 patients (64%) were at low-risk (LR), 202 (33%) at intermediate-risk (IR) and 21 (3%) at high-risk (HR) according to the 2015 ATA risk-stratification. Patients’ characteristics are reported in Table 1.

**Table 1:** Characteristics of patients according to the 2015 ATA risk-stratification system in the postoperative setting

	LR (*n* = 395)	IR (*n* = 202)	HR (*n* = 21)	*p*
Mean age ± SD (yrs)	49 ± 15	51 ± 18	67 ± 10	<.0001
Sex ratio (Female)	3.8 (79%)	2.0 (67%)	2.5 (71%)	0.005
Mean tumor size ± SD (mm)	22 ± 15	25 ± 18	51 ± 34	<.0001
Histology				
PTC	348 (88%)	169 (84%)	11 (52%)	<.0001
FTC	47 (12%)	12 (6%)	4 (19%)	
PDTC	0	21 (10%)	6 (29%)	
Aggressive pathological subtypes				<.001
No	395 (100%)	172 (85%)	18 (86%)	
Yes	0	30 (15%)	3 (14%)	
Extra-thyroidal extension				<.0001
Minimal	0	91 (45%)	1 (5%)	
Gross	0	0	14 (67%)	
T status (TNM 2017)				<.0001
T1a + T1b	230 (58%)	112 (56%)	1 (5%)	
T2	152 (39%)	57 (28%)	4 (19%)	
T3a + T3b	13 (3%)	33 (16%)	2 (9%)	
T4a + T4b	0	0	14 (67%)	
N status (TNM 2017)				<.0001
Nx	249 (63%)	31 (15%)	6 (28%)	
N0	119 (30%)	27 (13%)	5 (24%)	
N1a + N1b	27 (7%)	144 (72%)	10 (48%)	
M status (TNM 2017)				<.0001
M0	395 (100%)	202 (100%)	10 (48%)	
M1	0	0	11 (52%)	
Positive TgAb level	48 (12%)	32 (16%)	2 (10%)	0.43
Stimulated Tg level at RAI treatment (range)^a^	1.9 (0.1–744.0)	6.4 (0.1–4340.0)	126.2 (0.4–58,690.0)	<.0001

^a^In patients without positive TgAb level

### Persistent disease and tumor burden

Overall, PD was detected in 107/618 (17%) patients. Their characteristics in terms of ATA risk, RAI preparation modality, PD sites and RAI or ^18^FDG uptake are presented in Table 2.

**Table 2:** Characteristics of patients with persistent disease according to the tumor burden

	Very small-volume PD (*n* = 24)	Small-volume PD (*n* = 25)	Large-volume PD (*n* = 58)	*p*
Postoperative ATA risk				<.0001
LR	13 (54%)	5 (20%)	4 (7%)	
IR	11 (46%)	18 (72%)	37 (64%)	
HR	0	2 (8%)	17 (29%)	
Preparation modality				0.007
THW	9 (37%)	14 (56%)	42 (72%)	
rhTSH	15 (63%)	11 (44%)	16 (28%)	
PD site				0.002
LN	9 (38%)	17 (68%)	30 (52%)	
LN + DM	2 (8%)	2 (8%)	8 (14%)	
DM	13 (54%)	6 (24%)	9 (15%)	
TB disease	0	0	6 (10%)	
TB disease + DM	0	0	5 (9%)	
RAI and ^18^FDG status				<.0001
RAI+/^18^FDG- or NP	22^a^ (92%)	17^b^ (68%)	16^c^ (27%)	
RAI+/^18^FDG+	0	2 (8%)	12 (21%)	
RAI−/^18^FDG+	2 (8%)	6 (24%)	23 (40%)	
RAI−/^18^FDG-	0	0	6 (10%)	
RAI−/^18^FDG NP	0	0	1 (2%)	

^a^21 RAI+/^18^FDG NP and one RAI+/^18^FDG-

^b^15 RAI+/^18^FDG NP and two RAI+/^18^FDG-

^c^10 RAI+/^18^FDG NP and six RAI+/^18^FDG-

Of 107 patients, 24 (22%) had very small-volume, 25 (23%) small-volume and 58 (55%) large-volume PD.

**Fig. 2:**
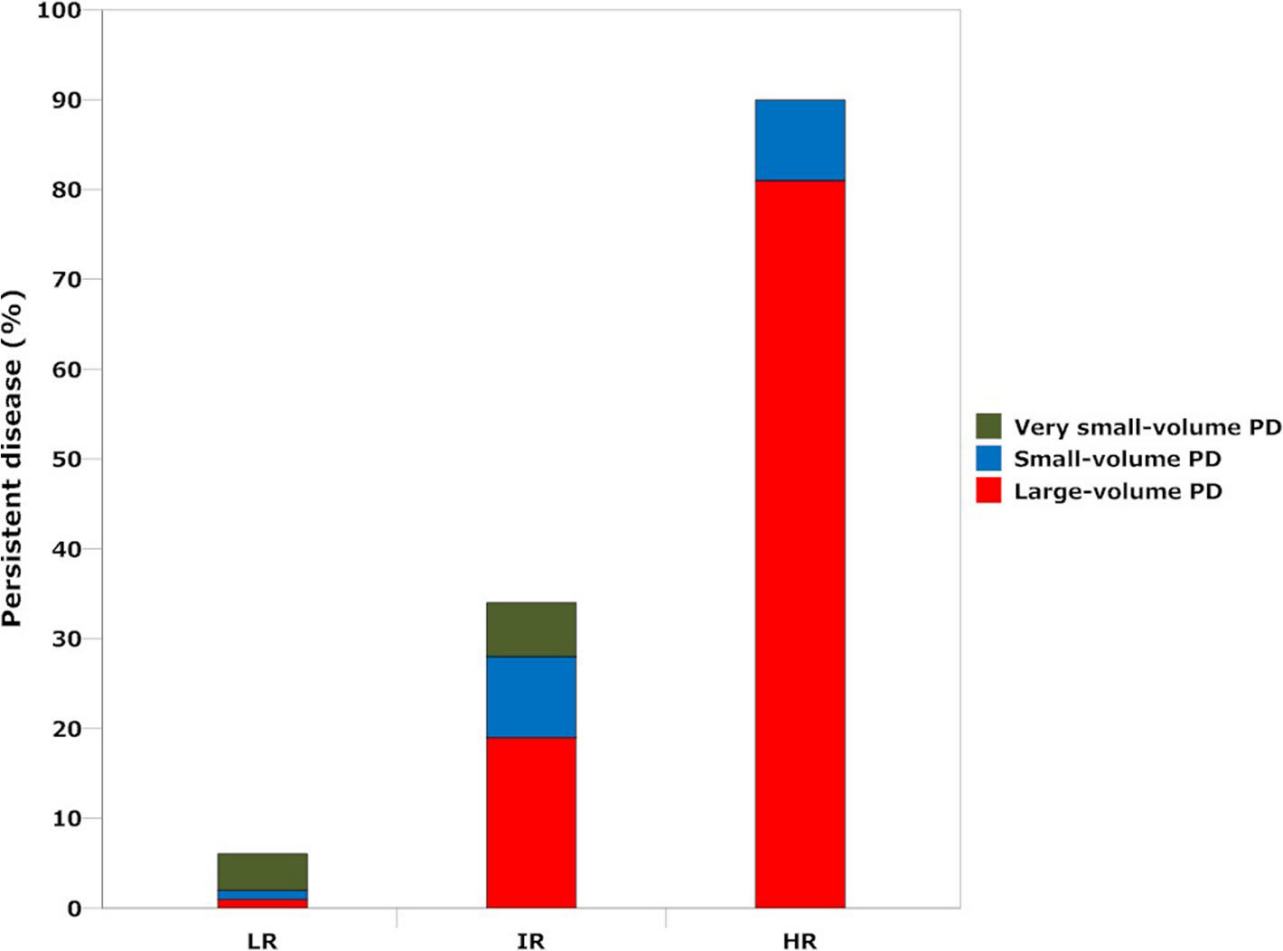
Tumor burden in patients with persistent disease: correlation to the 2015 ATA risk-stratification system. The figure first shows that the rate of PD increased from 6% in LR patients, 33% in IR to 90% in HR patients (*p* = 0.02). Second, the percentage of patients with large-volume PD increased with risk stratification from LR, IR to HR patients (18, 56 and 89%, respectively; *p* < 0.0001).

Figure 2 shows two points. First, the rate of PD increased from 6% (22/395) in LR patients and 33% (66/202) in IR to 90% (19/21) in HR patients (*p* = 0.02). Second, the percentage of patients with large-volume PD increased with risk stratification from LR, IR to HR patients (18, 56 and 89%, respectively; *p* < 0.0001). The distribution of very small-, small- and large-volume PD in LR, IR and HR patients is presented in Table 3.

**Table 3:** Characteristics of patients with persistent disease according to the 2015 ATA risk-stratification system

	LR (*n* = 22)	IR (*n* = 66)	HR (*n* = 19)	*p*
PD tumor burden				
Very small-volume	13 (59%)	11 (17%)	0	
Small-volume	5 (23%)	18 (27%)	2 (11%)	<.0001
Large-volume	4 (18%)	37 (56%)	17 (89%)	

### Outcome of patients with persistent disease

**Fig. 3:**
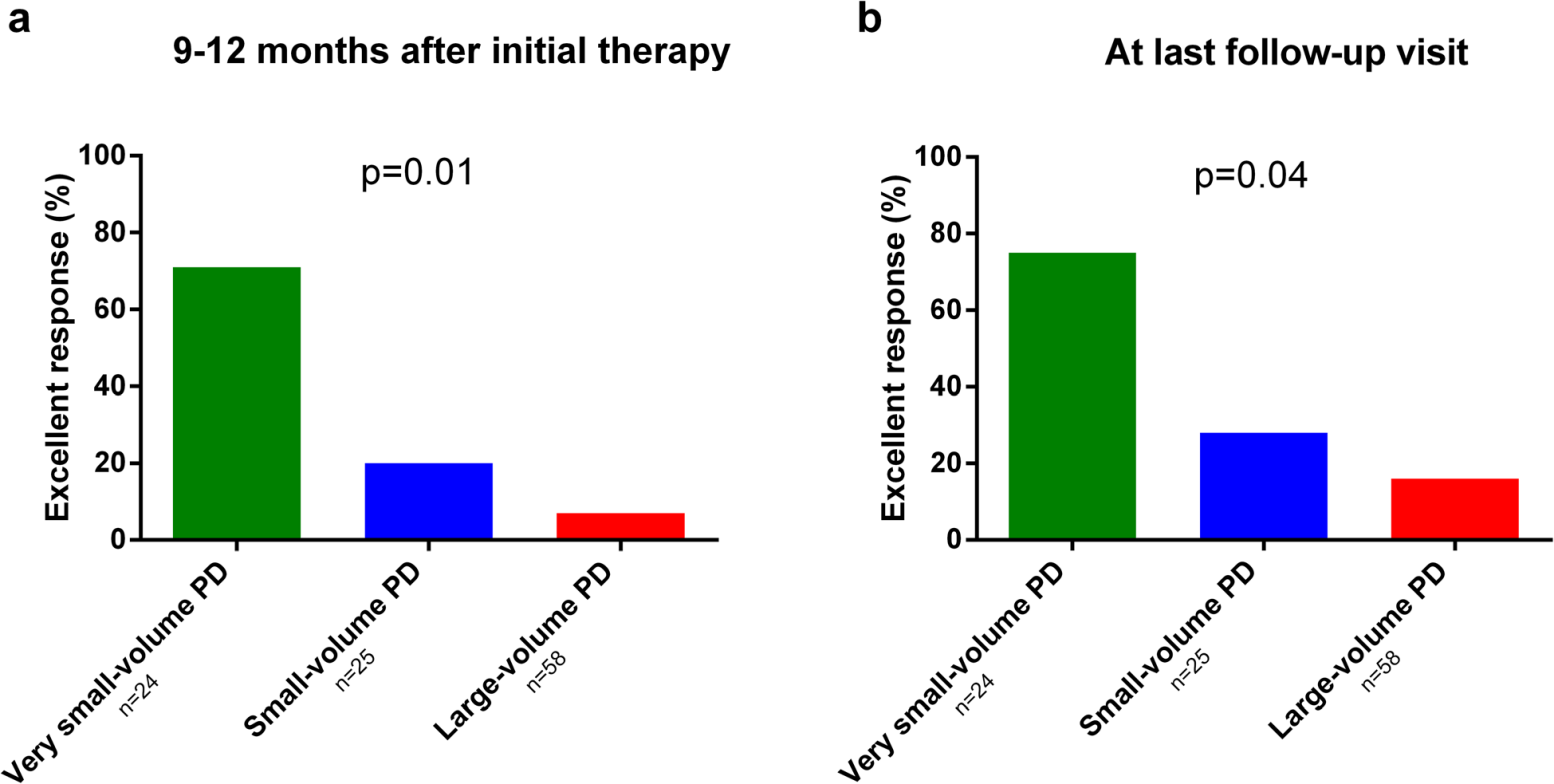
Excellent response rate according to tumor burden 9–12 months after initial therapy (**a**) and at last follow-up visit (**b**) in patients with persistent disease. There is a significant trend for a decrease in excellent response rate from the very small-, small- to the large-volume PD groups at 9–12 months after initial therapy (71, 20 and 7%, respectively; *p* = 0.01) and at last follow-up visit (75, 28 and 16%, respectively; *p* = 0.04).

Treatment modalities within the first year of management and during the remaining follow-up are detailed in Table 4. Mean follow-up for patients with PD was 7 ± 3 years and was similar between the three groups of tumor burden (*p* = 0.15). Of the 107 patients with PD, at 9–12 months after initial therapy, 26 (24%) had excellent response, 11 (10%) indeterminate response, 8 (8%) biochemical incomplete response and 62 (58%) structural incomplete response. At last follow-up visit, the figures were 34 (32%), 18 (17%), 17 (16%) and 38 (35%), respectively. The outcome in each of the tumor burden groups is presented in Table 4. There was a significant trend for a decrease in excellent response rate from the very small-, small- to the large-volume PD groups at 9–12 months after initial therapy (71, 20 and 7%, respectively; *p* = 0.01) and at last follow-up visit (75, 28 and 16%, respectively; *p* = 0.04) (Fig. 3).

**Table 4:** Treatment modalities and outcome of patients with PD at 9–12 months after initial therapy and at last follow-up visit according to tumor burden

		9–12 months after initial therapy				At last follow-up visit		
	Very small-volume PD (*n* = 24)	Small-volume PD (*n* = 25)	Large-volume PD (*n* = 58)	*p*	Very small-volume PD (*n* = 24)	Small-volume PD (*n* = 25)	Large-volume PD (*n* = 58)	*p*
Treatment modalities^a^								
RAI	24 (100%)	25 (100%)	58 (100%)		10 (40%)	4 (16%)	19 (33%)	
Neck surgery	0	2 (8%)	22 (39%)		0	5 (20%)	17 (30%)	
Neck external radiation beam therapy	0	0	7 (12%)		0	1 (4%)	6 (11%)	
Local treatment of DM^b^	0	0	7 (12%)		0	2 (8%)	13 (23%)	
Tyrosine-kinase inhibitors	0	0	0		0	1 (4%)	12 (21%)	
Chemotherapy	0	0	0		0	0	1 (2%)	
Outcome				< 0.0001				0.0003
Excellent response	17 (71%)	5 (20%)	4 (7%)		18 (75%)	7(28%)	9 (16%)	
Indeterminate response	2 (8%)	6 (24%)	3 (5%)		2 (8%)	6 (24%)	10 (17%)	
Biochemical incomplete response	2 (8%)	3 (12%)	3 (5%)		3 (13%)	4 (16%)	10 (17%)	
Structural incomplete response	3 (13%)	11 (44%)	48 (83%)		1 (4%)	8 (32%)	29 (50%)	

^a^ Treatment modalities at 9–12 months after initial therapy: treatments given within the first year of follow-up; treatment modalities at last follow-up visit: treatments given after the first year during follow-up

^b^ Local treatment of DM: external radiation beam therapy, surgery or radiofrequency

Abbreviations: *PD* Persistent disease; *RAI* Radioiodine; *DM* Distant metastases

Among the 107 patients, 8 (7%) died related to DTC during follow-up. Seven were in the large-volume PD group and one in the small-volume PD group. All had structural incomplete response at 9–12 months after initial therapy with ^18^FDG-positive disease.

**Fig. 4:**
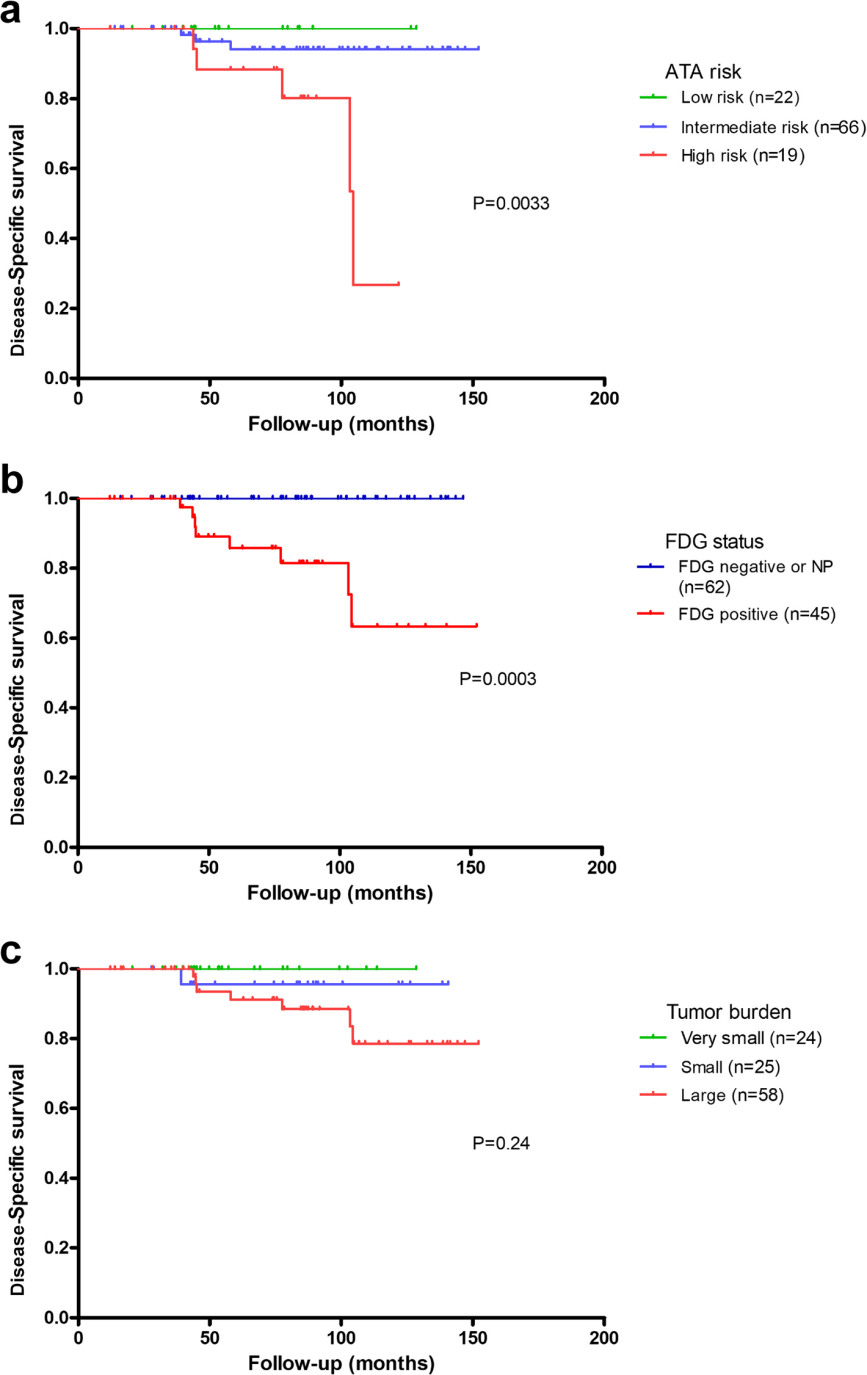
Disease-specific survival in the 107 patients with PD according to ATA risk-stratification (**a**), ^18^FDG status (**b**) and tumor burden (**c)**.

**Fig. 5:**
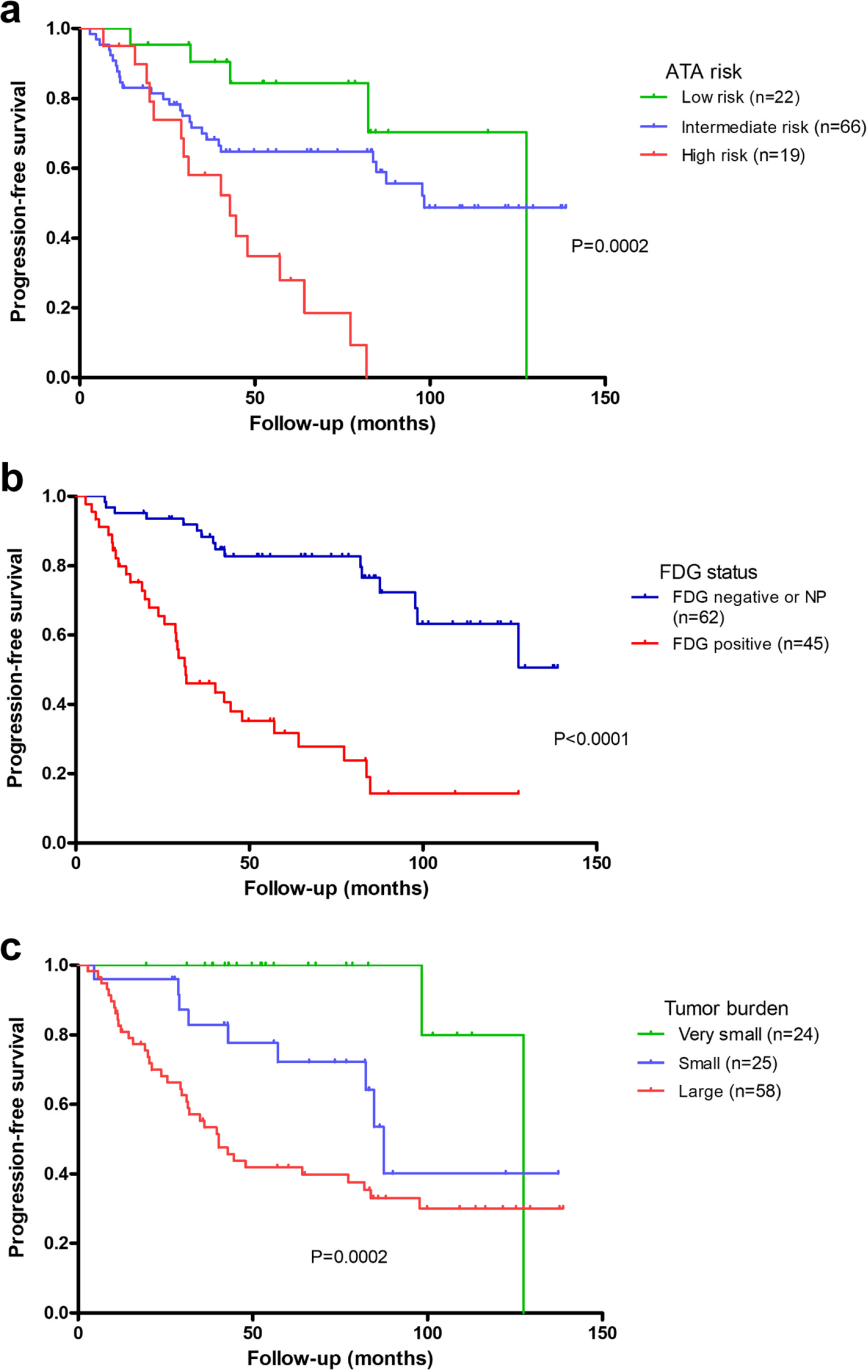
Progression-free survival in the 107 patients with PD according to ATA risk-stratification (**a**), ^18^FDG status (**b**) and tumor burden (**c**).

Figures 4 and 5 show disease-specific survival (DSS) and progression-free survival (PFS) according to the ATA risk-stratification, ^18^FDG status and tumor burden. Significant differences in DSS were observed for both ATA risk-stratification and ^18^FDG status, but not for tumor burden. Patients with ^18^FDG-positive disease had shorter PFS (Hazard Ratio = 5.1, 95%CI: 2.8–9.6) than those with ^18^FDG-negative disease. Also, IR (Hazard Ratio = 1.8, 95%CI: 0.7–4.7) and HR patients (Hazard Ratio = 5.4, 95%CI, 1.9–14.7) had shorter PFS than LR patients. Finally, patients with small- (Hazard Ratio = 4.6, 95%CI, 1.0–21.2) and large-volume PD (Hazard Ratio = 10.0, 95%CI, 2.4–41.4) had shorter PFS than those with very-small volume PD.

### Prognostic factor analysis in patients with persistent disease

Multivariate analysis controlling for age, sex, postoperative ATA risk-stratification, aggressive pathological subtypes, site of PD, tumor burden of PD and RAI or ^18^FDG uptake showed age ≥ 45 years (Odds ratio [OR], 3.8; *p* = 0.02), distant and/or thyroid bed disease (OR, 6.8; p = 0.02), small-volume (OR, 15.1; *p* < 0.01) and large-volume tumor burden (OR, 19.2; *p* < 0.001), and ^18^FDG-positive disease (OR, 8.7; *p* < 0.01) to be independent risk factors for indeterminate, biochemical or structural incomplete response at last follow-up visit (Table 5).

**Table 5:** Risk factors for indeterminate, biochemical or structural incomplete response at last follow-up visit

		Initial model			Final model		
Variable	Patients at risk, n	OR	95% CI	*p* value	OR	95% CI	*p* value
Age, years							
< 45	38	1.0			1.0		
≥ 45	69	5.3	2.2–12.8	<.001	3.8	1.2–11.9	0.02
Sex							
Female	69	1.0					
Male	38	1.5	0.6–3.6	0.37			
Initial 2015 ATA risk-stratification							
LR	22	1.0					
IR	66	5.7	2.0–16.3	<.01			
HR	19	38.6	4.2–349.5	<.01			
Aggressive histological subtypes							
No	82	1.0					
Yes	25	3.0	1.0–9.7	0.06			
Site of PD							
LN only	62	1.0			1.0		
DM and/or TB disease with or without LN	45	1.5	0.7–3.5	0.33	6.8	1.4–34.0	0.02
Tumor burden of PD							
Very small-volume	24	1.0			1.0		
Small-volume (< 10 mm)	25	7.7	2.2–27.5	<.01	15.1	2.6–89.3	<.01
Large-volume (≥10 mm)	58	16.3	5.1–52.4	<.0001	19.2	3.8–98.8	<.001
RAI and ^18^FDG status of PD							
RAI+/^18^FDG- or NP	55	1.0			1.0		
RAI−/^18^FDG- or NP	7	1.4	0.3–6.80	0.69	1.5	0.2–11.0	0.71
RAI- or RAI+/^18^FDG+	45	14.5	4.0–52.5	<.0001	8.7	1.8–41.9	<.01

## Discussion

This study confirms that the incidence of PD after total thyroidectomy and postoperative RAI treatment is limited in LR patients (6%) as compared to IR (33%) or HR patients (90%). Moreover, it demonstrates that the tumor burden of PD is correlated to postoperative risk-stratification with very small-volume lesions preferentially observed in LR patients and small and large-volume in IR or HR patients. Most importantly, tumor burden of PD is shown as an independent predictor of response to initial therapy and to outcome. These findings confirm that tumor burden of PD is a variable which might be taken into account to refine outcome prognostication.

Tumor burden covers a large range of loco-regional and/or distant metastases, from a unique microscopic lesion to multiple macroscopic ones, sometimes clinically evident. Also, tumor burden encompasses structural, e.g. visible on conventional radiology, and/or functional lesions, e.g. visible on RAI scintigraphy or ^18^FDG PET/CT. The diagnostic performances of imaging methods, and consequently, the concept of tumor burden, have dramatically evolved in the last decades. The detection of small LN disease has been improved by the combination of high-resolution neck US, post-RAI SPECT/CT and ^18^FDG PET/CT imaging. Regarding distant metastases, although post-RAI WBS still remains the reference for detecting lung miliary disease, the routine use of diagnostic CT scan and MRI now enables the detection of infracentimetric lung, bone or brain lesions.

In the past, tumor burden of PD as a potential indicator of successful treatment and prognosis was assessed using different approaches. In a study on 134 DTC patients with lung metastases diagnosed from 1967 to 1989, multivariate analysis showed that lung nodules visible on X-Ray (vs. those not visible), RAI-refractory lung lesions and multiple metastatic sites were associated with poor survival [8]. In Gustave Roussy’s experience, overall survival was reported in 444 DTC patients with distant metastases (lung, bone or other sites) diagnosed between 1953 and 1994 [2]. Tumor extent was classified into three categories according to both post-RAI planar scintigraphy and X-rays. Category 1 consisted in lesions visible on post-RAI scan but with normal X-ray, category 2 in metastatic lesions < 1 cm on X-rays and category 3 in lesions > 1 cm regardless of RAI avidity. Overall, metastases were RAI-avid in 68% of patients, more frequently in patients < 40 years (91%) than > 40 years (58%). Multivariate analysis demonstrated that female sex, young age (< 40 years), well differentiated tumor, RAI avidity and limited extent (category 1) were independent predictors of survival. More recently, Robenshtok et al. reported the outcome of 14 patients with RAI-avid bone metastasis without structural correlate on CT scan or MRI (among 288 DTC patients with bone metastases between 1960 and 2011) [13]. After a follow-up period of 5 years, all patients were alive, none had evidence of structural bone metastases, and none had experienced skeletal-related events, confirming the excellent prognosis after RAI treatment.

In DTC patients with persistent nodal disease, there is also indirect evidence supporting that tumor burden affects treatment response and outcome. In a recent retrospective study, Lamartina et al. reported the outcome of 157 patients without distant metastases who underwent a first neck reoperation for nodal persistent/recurrent disease [14]. Male sex, aggressive histology and the presence of more than 10 LN metastases at reoperation were shown to be independent risk factors of secondary relapse following complete response achieved with first reoperation. Conversely, the excellent outcome of microscopic nodal involvement detected on SPECT/CT at RAI ablation was demonstrated by a study from Schmidt et al. [15]. Of 20 patients with RAI-avid LN metastases at ablation, only three still showed nodes with significant uptake on a diagnostic RAI scintigraphy at 5 months. The LN successfully treated by RAI were less than 1 cm except in one patient whereas those still visible at 5 months were above 1 cm confirming that RAI is highly more efficient in microscopic than in macroscopic lesions.

In the present study, multivariate analysis showed that age over 45 years, distant and/or thyroid bed disease, small- or large-volume tumor burden and ^18^FDG-positive disease were independent risk factors for indeterminate or incomplete response at last follow-up visit. In contrast, ATA risk stratification and aggressive pathological subtypes did not emerge from multivariate analysis, possibly because of the number of patients, the number of variables tested and confounding variables. However, the disease-specific and progression-free survival curves confirmed the high prognostic value of the ATA risk-stratification. In practice, data supports that LR patients have a better outcome than the IR and HR groups not only because PD is uncommon in those patients, but also because the excellent response rate is higher in very small-volume than in small- or large-volume lesions. We suggest that tumor burden using this three-class discrimination could be implemented in the assessment of patients with structural incomplete response to help refining the risk prediction. This variable could also be incorporated with the other risk predictors such as RAI or ^18^FDG uptake, molecular profile, tumor histology, degree of cell differentiation, and Tg level and tumor volume doubling time, to further improve risk estimates.

Although retrospective, the present study presents several strengths including the large cohort of consecutive patients and the significant follow-up. Patients diagnosed between 2006 and 2016 were uniformly evaluated using modern imaging studies, including post-RAI scintigraphy with neck and thorax SPECT/CT [16] and ^18^FDG PET/CT with a dedicated head-and-neck acquisition [17, 18]. Tumor burden was assessed combining functional and anatomic imaging, as adapted from previous papers of our group [9, 19]. One can argue that it would have been even more pertinent to assess tumor burden with quantitative values rather than with a three-class discrimination (i.e., very small-, small- and large-volume). Actually, a quantitative volumetric assessment is not feasible because of the RAI-avid nodal or metastatic lesions without structural correlate. Also, a quantitative assessment based on RAI or ^18^FDG uptake is not possible either, because of RAI-refractory or non-hypermetabolic lesions. Nevertheless, we believe that our definition is simple to use in routine practice and easily reproducible.

## Conclusions

The tumor burden of PD correlates with the postoperative ATA risk-stratification, affects the response to initial therapy and is an independent predictor of residual disease after a mean 7-yr follow-up. This variable might be taken into account in addition to the postoperative ATA risk-stratification to refine outcome prognostication after initial treatment.

## Data Availability

The datasets used and analysed during the current study are available from the corresponding author on reasonable request.

## Abbreviations

ATA: American thyroid association
DM: Distant metastases
DTC: Differentiated thyroid cancer
^18^FDG: ^18^F-fluorodeoxyglucose
FTC: Follicular thyroid cancers
HR: High-risk
IR: Intermediate-risk
LN: Lymph-nodes
LR: Low-risk
MRI: Magnetic resonance imaging
NP: Not performed
OR: Odds ratio
PD: Persistent disease
PDTC: Poorly-differentiated thyroid cancers
PET/CT: Positron emission tomography with computed tomography
PTC: Papillary thyroid cancers
RAI: Radioiodine
rhTSH: Recombinant human thyrotropin
SPECT/CT: Single photon emission computed tomography with computed tomography
Tg: Thyroglobulin
TgAb: Anti-Tg antibodies
THW: Thyroid hormone withdrawal
TB: Thyroid bed
US: Ultrasound examination
WBS: Whole-body scan
